# Symptomatic extraforaminal gas-containing pseudocyst treated with unilateral biportal endoscopic spinal surgery: a case report and literature review

**DOI:** 10.3389/fsurg.2025.1521271

**Published:** 2025-03-19

**Authors:** Ya-wen Zhang, Bin Xu, Xu-ke Wang, Ao-te Zheng

**Affiliations:** ^1^ Department of Nursing, Tongde Hospital of Zhejiang Province, Hangzhou, Zhejiang, China; ^2^ Department of Orthopedics, Tongde Hospital of Zhejiang Province, Hangzhou, Zhejiang, China; ^3^ Department of Spinal Minimally Invasive, Luoyang Orthopedic Hospital of Henan Province, Orthopedic Hospital of Henan Province, Luoyang, Henan, China; ^4^ Department of Anesthesiology, Tongde Hospital of Zhejiang Province, Hangzhou, Zhejiang, China

**Keywords:** gas-containing pseudocys, extraforaminal area, unilateral biportal endoscopic spinal surgery, case report, literature review

## Abstract

**Background:**

Gas-containing pseudocyst is an uncommon cause of lumbar radiculopathy and most lumbar gas-containing pseudocysts locate in the spinal canal. While, extraforaminal gas-containing pseudocysts are very rare. Here, we reported a case of extraforaminal gas-containing pseudocyst, which compressed L4 exiting nerve root and caused lumbar radiculopathy.

**Case presentation:**

A 62-year-old female presented with low back pain and radiation to anteromedial aspect of right thigh and anterior aspect of right calf. Computed tomography and magnetic resonance imaging of lumbar spine showed a gas-containing pseudocyst compressing in L4 exiting nerve root right extraforaminal area at L4–5 level. L4 exiting nerve root blocking was performed to confirm the responsible level. Then we performed BESS through a paraspinal approach to remove the gas-containing pseudocyst and release L4 exiting nerve root. Postoperatively, the patient achieved a good outcome and the pain was relieved.

**Conclusions:**

Lumbar gas-containing pseudocyst in extraforaminal area is rare and can cause lumbar radiculopathy. Paraspinal approach BESS is an alternative method to treat extraforaminal gas-containing pseudocyst and can provide good outcome.

## Introduction

Epidural gas-containing pseudocysts (GCPs) are uncommon causes of lumbar radiculopathy and it has been reported that GCPs are often associated with the intervertebral vacuum phenomenon (1–3). Most lumbar GCPs locate in the spinal canal and coexist with a disk fragment (4, 5). While the extraforaminal GCPs causing nerve root compression are extremely rare (3–5).

The treatment of lumbar GCPs including conservative management, percutaneous needle aspiration, percutaneous endoscopy and open surgery (6, 7). As a new endoscopic technique, unilateral biportal endoscopic spinal surgery (BESS) increases surgical movement of instruments with the independent visualization and working portals, provides good and wide field of visualization to unrestricted access contralateral and foraminal and extraforaminal areas (3). BESS has been applied to treat lumbar disc herniation and lumbar spinal stenosis (4). In this report, we present a symptomatic extraforaminal GCP, which was removed by BESS.

## Case presentation

**Figure 1:**
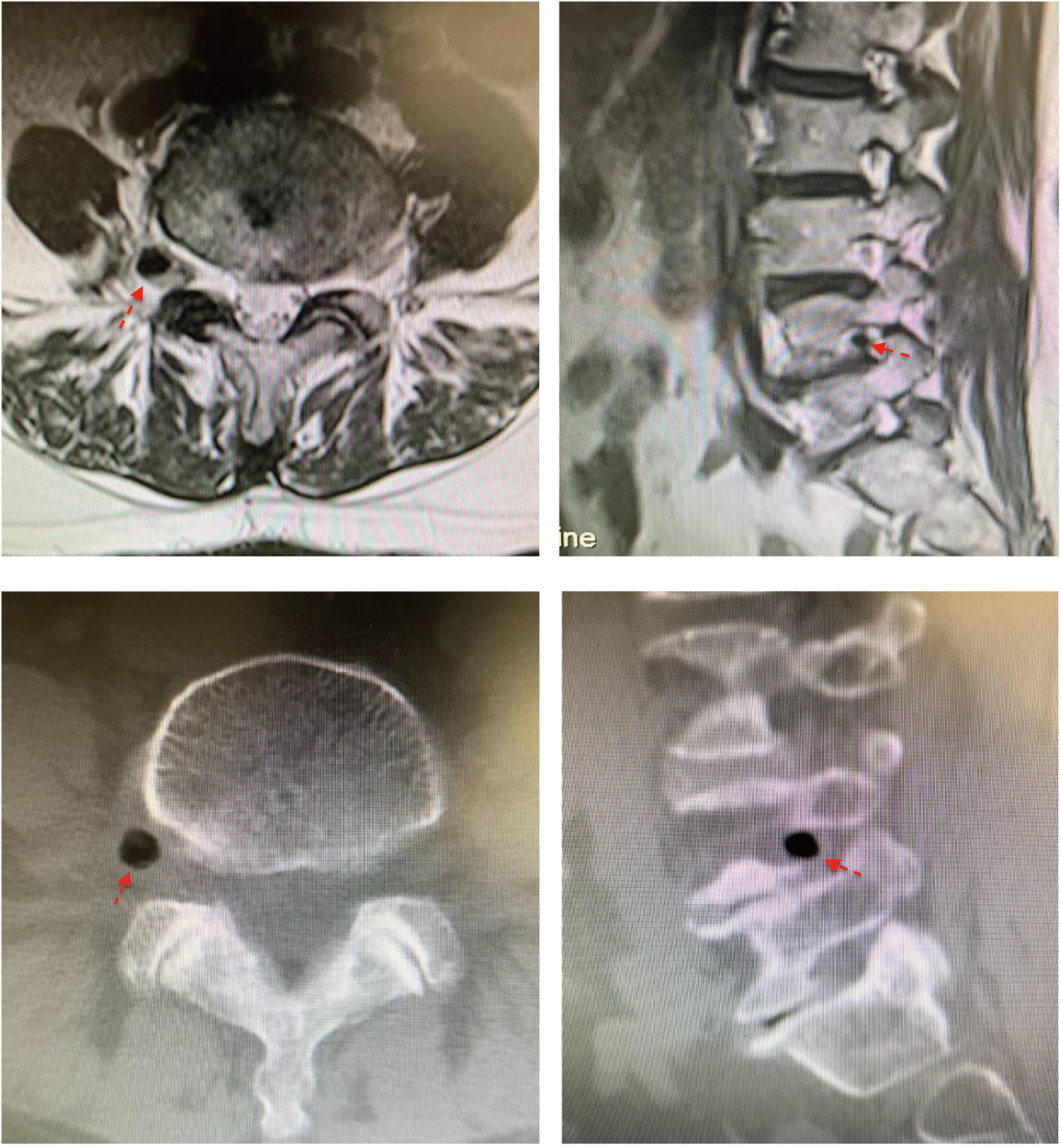
Right extraforaminal GCP of L4–5 was shown in MRI and CT. **(A)** MRI axial views of L4–5; **(B)** MRI sagittal view of L4–5; **(C)** CT axial views of L4–5; **(D)** CT sagittal view of L4–5. Red arrow indicated the extraforaminal GCP.

A 62-year-old female presented with low back pain and radiation to anteromedial aspect of right thigh and anterior aspect of right calf. The VAS score was 6 and the pain had persisted for 3 months. Physical examination revealed a positive straight leg raising (SLR) sign and lasegue test on the right side. Magnetic resonance imaging (MRI) and computed tomography (CT) of lumbar spine showed a right extraforaminal GCP in L4–5 level, which compressing L4 exiting nerve root (Figure 1).

Before BESS, we performed L4 exiting nerve root blocking in the extraforaminal area. The patient reported obvious pain relief after this blocking, which indicated that the responsible level was L4/5, and the target was the right extraforaminal GCP in L4–5 level.

**Figure 2:**
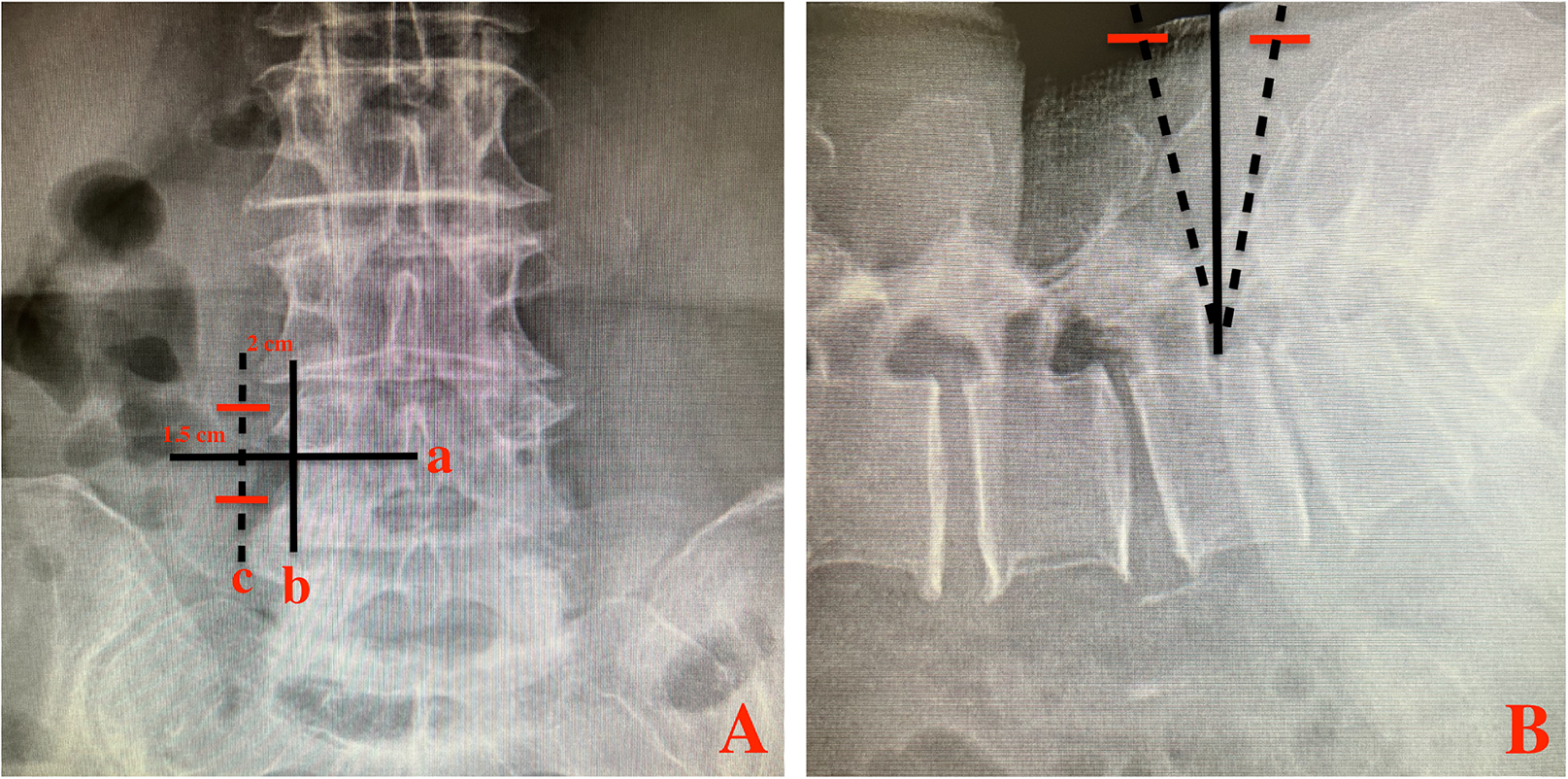
Two transverse skin incisions (red lines) made on the fluoroscopic AP **(A)** and lateral **(B)** views. line a: inferior margin of L4 pedicle; line b: external margin of L4 pedicle; line c: parallel line of b; Red line: skin incision.

We performed BESS through a paraspinal approach. The patient was placed in prone position on a radiolucent table and under general anesthesia. After the target level was confirmed under fluoroscopic guidance, the skin entry point was 1.5 cm above and 1.5 cm below the inferior margin of L4 pedicle, and 2 cm lateral to external margin of L4 pedicle (Figure 2). Two portals (walking portal and viewing portal) were created according to the skin entry point.

**Figure 3:**
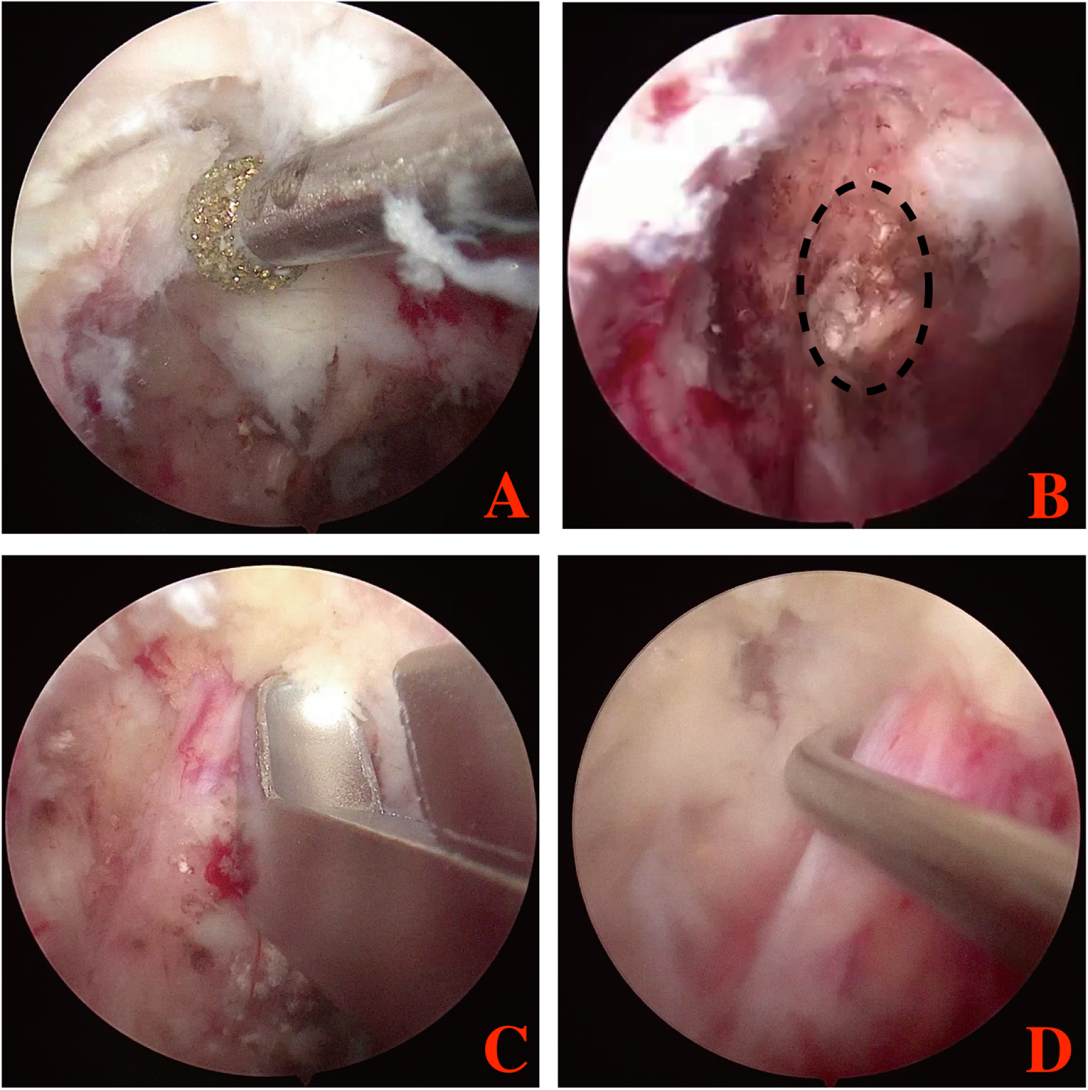
Endoscopic image during the BESS procedure. **(A)** the extraforaminal area was exposed; **(B)** extraforaminal GCP was exposed and removed; **(C,D)** L4 exiting nerve root were exposed and released. Black dotting circle: the extraforaminal GCP.

**Figure 4:**
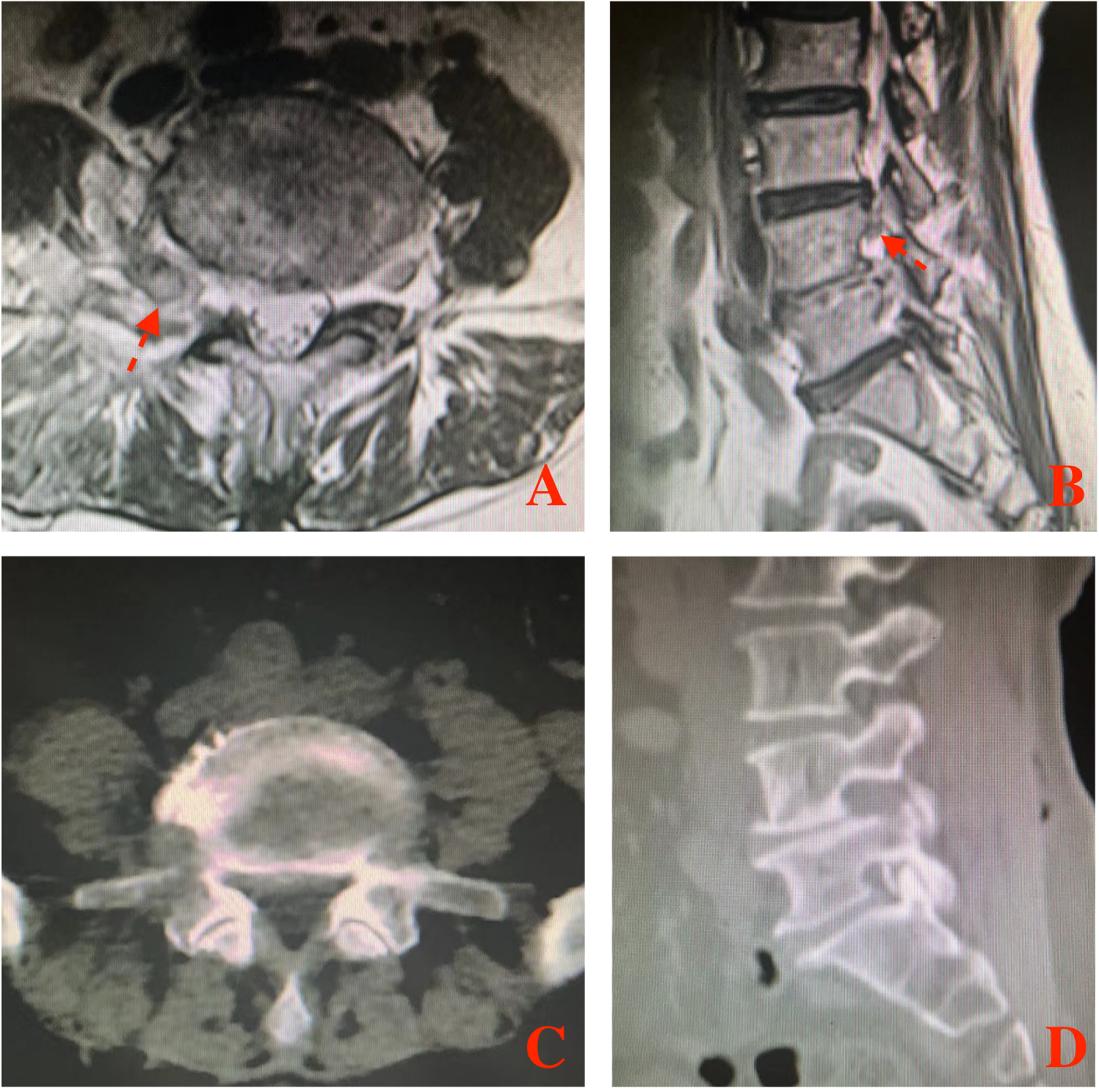
The extraforaminal area was shown in postoperative MRI and CT. **(A)** MRI axial views of L4–5; **(B)** MRI sagittal view of L4–5; **(C)** CT axial views of L4–5; **(D)** CT sagittal view of L4–5. Red arrow indicated the L4 exiting nerve root.

Serial dilators were used to dissect the back muscle and acquire operative space. The trocar of the scope was introduced into the viewing portal and a round, smooth periosteal elevator was inserted into the working portal. After triangulation occurs between scope and the periosteal elevator, minor bleeding was controlled and remnant soft tissues were removed by radiofrequency probe and shaver, to expose L5 superior articular process, L4 inferior articular process, L4 pedicle isthmus and the base of L4 transverse. Then, part of L4 transverse and pedicle isthmus, the tip of L5 superior articular process and the exterior part of L4 inferior articular process were removed with a 3 mm drill or arthroscopic burr and Kerrison punch (Figure 3). After that, the ligament flavum in foramen was exposed and flavectomy was performed. Maneuvering of the scope, extraforaminal GCP, exiting nerve root and foramen would be directly visual­ized (Figure 3). Through the working portal, pituitary forceps were used to remove the extraforaminal GCP, and the L4 exiting nerve root was successfully decompressed (Figure 3). The successful removal of extraforaminal GCP was confirmed on postoperative MRI and CT (Figure 4). A bone tunnel could be seen on a CT scan (Figure 4). The patient achieved a good outcome, VAS score improved to 3 on the first postoperative day, and improved to 1 on 7 days after operation.

## Discussion

In the spine, the presence of gas or vacuum phenomenon is a relatively common radiological finding, and generally occurs in the intervertebral disc spaces (8). Compared with intervertebral gas, epidural GCP is much rare (7). Kuh et al. assessed the reported that 48.0% epidural GCP were found in the canal, 32.0% were found in the foramen, and 20.0% were found in extraforaminal area (9). We reviewed the articles about epidural GCPs, and found that 20 articles reported 43 cases of epidural GCPs (Table 1). Among them, only 9 cases (20.9%) of epidural GCPs located in extraforaminal area (4, 6, 9).

**Table 1:** Case of radiculopathies caused by a spontaneous gas-containing pseudocyst.

Author	Sex	Age (years)	Symptoms duration	Spinal location	Compressed root	Treatment	Outcome
Bosser et al. (1990) (11)	F	62	6 months	L5 vertebrae	Right L5 root	Percutaneous needle aspiration, open surgery	Transitory resolution of radicular symptoms after needle aspiration but recurred; pain disappeared after open surgery
An et al. (1993) (5)	M	75	2 years	L5–S1	left L5 root	Unilateral biportal endoscopy	Remained asymptomatic 6 months later
Lin et al. (1994) (14)	M	40	6 months	L3 vertebrae	Right L3 root	Open surgery	/
Heissler et al. (2005) (15)	F	42	/	L4–5	right L5 root	Percutaneous needle aspiration	Remained asymptomatic at 1 year follow-up.
Lee et al. (2010) (16)	F	67	10 days	L2–3	Right L2 root	Open surgery	Pain was significantly improved after surgery
Yasuoka et al. (2010) (17)	M	48	1 year	L4–5	left L5 root	Surgical needle decompression	Pain relief; remained asymptomatic 9 months later.
Kuh et al. (2011) (9)	6 M 16F	67.6 ± 10.8	/	L2/3: 2 L3/4: 1 L4/5: 15 L5/S1: 7	/	Open surgery: 12 microscopic surgery: 10	All symptoms of neurologic compromise improved after surgery
Vaquero et al. (2011) (18)	M	65	/	L5-S1	Right S1 root	Open surgery	Completely free of symptoms during 6 years follow-up
Kim et al. (2011) (2)	F	67	3 days	L5–S1	left L5 root	Open surgery	Improvement of motor power; gradual recovery of the sensory deficits by 6 months
Pak et al. (2011) (4)	M	83	Acute	L5-S1	Left L5 root	Percutaneous needle aspiration	Remains symptom free after 6 months
Seo et al. (2012) (3)	F	69	4 months	L3–4 L4–5	Right L3, L4 root	Open surgery	No symptoms or complications during 6 months follow-up
Yun et al. (2012) (19)	M	83	8 weeks	L4–5	Left L5 root	Microsurgery	No remarkable complaints during a 6 month follow-up
	F	72	1 months	L5–S1	Left S1 root	Open surgery	No recurrence of complaint at 1-year follow up
Kang et al. (2012) (8)	F	68	3 years	L5–S1	Right S1 root	Epidural block and percutaneous needle aspiration	Almost complete resolution of radiating pain one year later
El Beltagi et al. (2013) (20)	F	51	3 months	L4–5	Right L4 and L5 root	Medications	/
Belfquih et al. (2014) (10)	F	45	9 months	L5–S1	Right S1 root	Open surgery	Remains free of pain at 1-year follow up
Zhu et al. (2017) (7)	M	57	1 year	L5–S1	Right S1 root	Percutaneous endoscopy	Remained pain-free at the last follow-up
Ferjani et al. (2021) (6)	F	78	1 year	L4–5	Right L4 root	Medications and rehabilitation	/
	/	61	2 months	L5–S1	left L5 root	Conservative management	Symptoms improved
Chen et al. (2021) (21)	M	78	3 weeks	L5–S1	Right S1 root	Percutaneous endoscopy	Complete relief of pain
Hu et al. (2022) (12)	F	59	2 years	L5–S1	Right S1 root	Conservative therapy percutaneous endoscopy	No improvement after conservative therapy pain-free 6 months after the surgery
Krishnan et al. (2022) (1)	F	50	Sudden	L5–S1	Right S1 root	Medications and rest	Radiculopathy decreased

In those reported cases, most epidural GCPs were found because of neurological symptoms, such as sciatica, lower extremity paresthesia and paralysis (6). The clinical features are very similar to common lumbar disc herniation. So CT and MRI are very helpful to diagnose and assess the epidural GCPs. Epidural GCPs can be identified with density from - 200 to - 900 Hounsfield units in CT scan (10), or with low signal on T1- and T2-weighted images of MRI (9). Sometimes, calcification has the same low signal intensity in MRI, making it difficult to distinguish between gas and calcification. So CT scan is the most useful radiological method for identifying epidural GCPs.

There are various therapeutic strategies to treat symptomatic epidural GCPs, including medications, percutaneous needle aspiration, surgical removal (endoscopic spinal surgery or open surgery) (5). The conservative management (such as medications and rest) and percutaneous needle aspiration are common treatments for epidural GCPs, especially for those patients with surgery contraindication. However, some authors reported that epidural GCPs recurred with a relapse of the radicular syndrome after conservative management or percutaneous needle aspiration (11, 12). Open surgery has good clinical outcomes by totally removing the epidural GCPs and herniated disk fragment. However, open surgery has large operative injury, as its protocol includes discectomy, decompressive laminectomy facetectomy and interbody fusion. So open surgery may be more suitable for those patients with concomitant spinal stenosis or segmental instability (9). Compared with open surgery, endoscopic spinal surgery has many surgical advantages, including less invasive procedure, faster postoperative recovery and lower cost (9). So for those patients without severe spinal stenosis or segmental instability, endoscopic spinal surgery may be a better choice. Percutaneous endoscopic surgery (interlaminar approach or transforaminal approach) is the most classical minimally invasive surgery for lumbar degenerative diseases. In classical percutaneous endoscopic surgery, a single portal was used, with multiple channels for irrigation, instrumentation, visualization, and a light source. So the single portal limited the motion of the instruments and obscures visualization of the operating field. Compared with classical percutaneous endoscopic surgery, BESS increases surgical movement of instruments with the independent visualization and working portals, provides good and wide field of visualization to unrestricted access contralateral and foraminal areas (13). So for those epidural GCPs located in lateral recess, foraminal or extraforaminal areas, BESS may be a better choice. In this case, we successfully removed extraforaminal gas-containing pseudocyst using BESS, and completely relieved the radiating pain.

## Conclusion

Lumbar GCPs in extraforaminal area is rare and can cause lumbar radiculopathy. Paraspinal approach BESS is an alternative method to treat extraforaminal gas-containing pseudocyst and can provide good outcome.

## Data Availability

The original contributions presented in the study are included in the article/Supplementary Material, further inquiries can be directed to the corresponding authors.
